# Isoflavone and Antioxidant of Instant Cream Soup Made from Pumpkin and Tempeh and Their Active Compound in Ovariohysterectomy Rat-Induced Alzheimer's Disease

**DOI:** 10.1155/2022/8051624

**Published:** 2022-11-28

**Authors:** Budi Setiawan, Salma S. Aulia, Ahmad Sulaeman, Clara M. Kusharto, Ekowati Handharyani

**Affiliations:** ^1^Department of Community Nutrition, Faculty of Human Ecology, IPB University, Bogor, Indonesia; ^2^Nutrition Study Program, Graduate School, IPB University, Bogor, Indonesia; ^3^Department of Veterinary Clinic Reproduction and Pathology, Faculty of Veterinary Medicine IPB University, Bogor, Indonesia

## Abstract

Menopause period associated with brain function disorders can caused by decreasing estradiol levels and increasing oxidative stress in the body. Antioxidant agents are required to balance oxidative stress in the body. Instant cream soup made from pumpkin and tempeh is a supplementary food containing isoflavone and antioxidant agents. This study is aimed at analyzing the content of antioxidant level and isoflavones (genistein and daidzein) in instant cream soup and their effect on ovariohysterectomy (OVx) rats. Instant pumpkin cream soup with and without tempeh were subjected to isoflavone content and antioxidant analysis. Serum estradiol was analyzed using enzyme-linked immunosorbent assay. Liver concentration of malondialdehyde (MDA) and activities of superoxide dismutase (SOD) were measured by spectrophotometric methods. The study showed that the isoflavone content (genistein 370.86 g/100 g, daidzein 185.61 g/100 g) was only present in the pumpkin instant cream soup with tempeh (IPTS). IPTS has higher antioxidant levels (134.25 mg AEAC/100 g) than instant pumpkin cream soup without tempeh (IPS). In vivo study, experimental rats showed that OVx increased malondialdehyde (MDA) levels up to 5.85-6.07 nmol mL^−1^ as compared to control (4.47 nmol mL^−1^). Moreover, instant pumpkin cream soup with tempeh treatments significantly increased serum estradiol levels (2.37–3.63 *μ*g) and superoxide dismutase (SOD) levels of 497.49-558.89 U mL^−1^. This study concluded that instant pumpkin cream soup and tempeh contained isoflavone and antioxidant, and it increased estradiol serum and SOD level.

## 1. Introduction

Menopause is the discontinuous of menstrual periods after 12 months of amenorrhea [1]. In menopause or perimenopause phase, the brain function related to cognitive function and working memory had changed. Research study showed that changes in brain function are related to the level of estradiol in the body [2]. Estradiol is estrogen hormone form involved in physiological processes such as cognitive function, mood regulation, learning ability, and memory [3, 4]. Estradiol also plays a vital role in the emergence of neurodegenerative diseases such as Alzheimer's, dementia, and stroke [5]. Beside the role of estradiol, the brain function can be affected by oxidative stress [6]. Oxidative stress is raised due to an imbalance in the number of reactive oxygen species (ROS) in the body and the body's ability to neutralize ROS [7]. To balance this oxidative stress, antioxidant agents are needed. Antioxidants can inhibit ROS synthesis by forming metal ion complexes involved in ROS catalysis [8].

Pumpkin (*Cucurbita moschata*) is a vegetable that offers many health benefits for humans [9]. Pumpkin was rich in carbohydrates, carotenoids, polyphenols, tocopherols, polysaccharides, pectin, inositol, minerals, and low in calories (15-25 kcal/100 g) [10]. Pumpkin also had good an antioxidant content [11–15]. Despite of these benefit, pumpkin has a low protein content [16]. One of the foods with high protein content is tempeh. Tempeh is an original Indonesian fermented food made from soybeans [17]. Besides having high protein, tempeh is also rich in isoflavones (genistein and daidzein). Isoflavones acted as antioxidants that can reduce oxidative stress [18–20]. The study of Cancellieri et al. [21] and Messina [22] showed good antioxidant activity of isoflavones in reducing the formation and progression of diseases such as Alzheimer's disease in menopause to several types of cardiovascular disease.

Our previous study has formulated an instant cream soup made from pumpkin and tempeh that is easy to consume, has a long shelf life, and has high nutritional content [16, 23]. Compared with other forms of food product, instant cream soup has longer shelf-life, is easier to prepare, preferable in modern society, and cheaper [24].

This study is aimed at analyzing the isoflavone content (genistein and daidzein) in instant cream soup and its antioxidant activity and analyze the effect of instant cream soup on estradiol levels, malondialdehyde, and superoxide dismutase in ovariohysterectomy rats.

## 2. Materials and Methods

### 2.1. Instant Cream Soup Formulation

The main ingredients of instant pumpkin cream soup and tempeh are pumpkin (*Cucurbita moschata* variety) and tempeh (Grobogan variety). The other ingredients for soup are carrots, leeks, onions, and rice flour. The procedure of pumpkin cream soup production was that the pumpkin was cut and cleaned from the seeds then sliced into cubes, while tempeh and carrots were cut into cubes, along with tiny slices for other ingredients. The onions were sautéed and the stock was poured. The pumpkin, tempeh, carrot, and leek were added. Then, the soup was boiled for 15 minutes. After the soup was cooked, it was mixed using a blender to become a puree. Cooking cream was added into the puree until mixed well, and rice flour was added after the cooking cream. Furthermore, the puree was dried using a drum dyer (120°C) for 60 seconds to produce cream soup powder [23].

### 2.2. Isoflavone Analysis

Instant cream soup was mixed with HCL 1 N: acetonitrile (1 : 4), stirred for 2 hours, and filtered with Whatman No.1. The sample-containing isoflavones were diluted 10 times with methanol as a mobile phase: ammonium acetate (6 : 4). daidzein and genistein standards in seven concentrations (from 0.5–50 g mL^−1^) were prepared as standards. The sample and standard solutions were filtered using a 0.22 *μ*m nylon membrane syringe filter. 20 *μ*L was injected into the HPLC (Agilent 1200 Series) using a 4.60 × 150 mm (5 *μ*m) Zorbax eclipse XDC-C18 column with a gradient elution system (methanol and ammonium acetate 6: 4) and a flow rate of 0.5 mL min^−1^. Detection was carried out with a UV-Vis multiwavelength detector at 265 nm [25].

### 2.3. Antioxidant Analysis

The instant cream soup was mixed with 10 mL of 80% methanol. The sample suspension was homogenized for 1 minute and centrifuged for 45 minutes at 3000 rpm, 4°C. The supernatant was separated, and an antioxidant analysis was performed. 0.1 mM DPPH solution was prepared in methanol and ascorbic acid (vitamin C) standard solution at 25–200 g mL^−1^. Antioxidant analysis was performed by mixing 0.2 mL of supernatant or ascorbic acid (as standard) with 3.8 mL of DPPH solution in a dark tube. The solution mixture was incubated at 30°C for 30 minutes in the dark. After that, absorbance measurements were taken using a UV-Vis spectrophotometer at 517 nm [26]. The percentage inhibition value was determined using

(1) $$\begin{array}{c} \text{DPPH radical scavenging activity }\left(\%\right)=\frac{\text{Ac}-\text{As}}{\text{Ac}}\times 100. \end{array}$$

Ac is the control DPPH absorbance, and As is the DPPH absorbance in the presence of extract/standard. The antioxidant capacity value is expressed in units of mg AEAC (ascorbic acid equivalent antioxidant capacity)/100 g instant cream soup.

### 2.4. Preparation of Experimental Animals

This study used female Sprague-Dawley rats^8^ aged 10 months with a bodyweight of 250-300 g from PT Biomedical Technology Indonesia. Rats were placed in an animal facility at the Animal Hospital (Rumah Sakit Hewan Pendidikan) IPB University and acclimatized for 14 days in individual cages with standard environmental conditions (temperature 26 ± 3°C and relative humidity 55–60%) and a 12-hour light-dark cycle. The research design and method have been approved by the Ethics Committee of the Institute for Research and Community Services (Lembaga Penelitian Pengabdian kepada Masyarakat) IPB University number 200-2021.

### 2.5. Ovariohysterectomy Animal Modeling

Ovariohysterectomy (OVx) was a method of animal sterilization as a model of menopause in humans [27]. Rats were anesthetized using a ketamine/xylazine mixture (ketamine 95 mg kg body weight and xylazine 5 mg kg body weight). OVx treatment was performed on the ventral abdomen. Abdominal hairs were incised by shaving bilaterally, and the skin was cleaned using alcohol and betadine antiseptic solution. After that, a 1–2 cm ventral midline incision is made at the navel location to expose the ventral abdominal muscles. The ovaries are excised, and all blood vessels and ducts are placed back into the abdomen before suturing. After the OVx process was completed, the rats were given analgesics, antibiotics, and paracetamol for five days for postoperative recovery.

### 2.6. Distribution of Treatment Groups

Rats were divided into four treatment groups: (a) control rats with standard feed (control), (b) OVx rats with standard feed (OH), (c) OVx rats with instant pumpkin cream soup feed (IPS), and (d) OVx rats with instant pumpkin cream soup enriched with tempeh (IPTS). The standard feed used AIN 93 M. The intervention feed using instant pumpkin cream soup and pumpkin instant cream soup enriched with tempeh [23]. Each rat was given drinking water ad libitum.

### 2.7. Serum Estradiol Analysis

The experimental rats with an intervention period of 60 days were taken under anesthesia with 10% ketamine (100 mg kg^−1^) and 2% xylazine (10 mg kg^−1^) by cardiac puncture method into plain tubes (red cap). The blood samples obtained were then centrifuged at 2500 rpm for 10 minutes at 25°C to obtain serum. Serum estradiol analysis was performed using the Rat E2 (estradiol) ELISA kit ELK Biotechnology (Catalog no: ELK8714). The estradiol (E2) concentration was determined by comparing the absorbance results at a wavelength of 450 nm between samples with a standard curve.

### 2.8. Superoxide Dismutase and Malondialdehyde Analysis

Analysis of superoxide dismutase (SOD) and malondialdehyde (MDA) was carried out using the liver of experimental rats. A total of 100 mg of rat liver was weighed and added phosphate buffer saline (PBS) 0.01 M. The mixture was then homogenized and centrifuged at 3500 rpm for 10 minutes. The supernatant was separated for SOD and MDA analysis. SOD analysis was performed using Ransod Superoxide Dismutase Manual Rx Monza Kit Randox (Catalog No: SD 125). The color change was measured using spectrophotometry at a wavelength of 505 nm. MDA analysis used the Wills (1987) method. The color change was measured using spectrophotometry at a wavelength of 530 nm.

### 2.9. Data Analysis

Data analysis was performed using Microsoft Excel 365 and analyzed with SPSS 25. Analysis of diversity used a different *T*-test and one-way ANOVA with Duncan's follow-up test (*p* < 0.05).

## 3. Results and Discussion

### 3.1. Potential Antioxidant Activity

Isoflavones was a group of phenolic compounds commonly found in soybeans and their processed products [28]. This compound has two forms, glycone and aglycone, in which the aglycone form is more easily absorbed by the body and enters the blood vessels [29]. The aglycone forms of isoflavones are daidzein, genistein, and glycitein [29]. Based on the results of the analysis, it is known that the pumpkin instant cream soup with tempeh (IPTS) contains high isoflavones (370.86 g genistein and 185.61 g daidzein), while the pumpkin instant cream soup without tempeh (IPS) does not contain isoflavones (Table 1). The high isoflavone content in IPTS is due to the addition of tempeh. Tempe has a genistein content of 30.8 mg/100 g [30]. Another study from Kridawati et al. [31] showed that tempeh has a high isoflavone (genistein) content of 50.56 mg/100 g. This content is twice as much as tofu flour which only contains 19.92 mg 100 g^−1^ of isoflavones.

Various diseases are caused by oxidative stress, such as Alzheimer's in menopausal women [18]. Oxidative stress can be prevented and minimized with the role of antioxidant agents. Cancellieri et al. [21] and Messina [22] show that antioxidant agents can reduce oxidative stress to reduce the formation and development of Alzheimer's disease in menopausal conditions to several types of cardiovascular disease. The analysis results showed that IPTS had the highest antioxidant activity, with an activity of 134.25 mg AEAC 100 g^−1^ (Table 1). The high antioxidant activity in IPTS is due to the synergism of compounds in pumpkin and tempeh, which are antioxidants such as carotenoids and phenolic compounds, especially isoflavones, vitamin E, and bioactive compounds peptides [32, 33]. Carotenoids are efficient antioxidants to protect cells from free radicals and single oxygen [34]. The study of Astawan et al. [25] and Chang et al. [35] showed that in vitro with the DPPH method, tempeh had an antioxidant activity of 169–203 mg AEAC 100 g^−1^ dry weight, while in vivo tempeh had an antioxidant activity with IC_50_ of 21.11–49.84 mg mL^−1^.

### 3.2. Estradiol Serum in Experimental Animals

Estradiol is the most common form of the hormone estrogen in the body and modulates cognitive function [36]. The decrease in estradiol in the body causes changes in gene transcription that result in an increased risk of cognitive impairment [37]. More significant effects such as decreased memory and learning function may occur when decreased estradiol is present in postmenopausal women [38, 39].

The analysis results showed that the control group and the OH group had lower estradiol than the IPS and IPTS groups (Table 2). These results are similar to previous studies that there was decrease in serum estrogen in OVx rat aged 12 months compared to non-OVx rat [40]. Another study showed that serum estradiol decreased in the postmenopause phase [2]. OVx was an ovarian removal treatment that describes postmenopausal conditions. This treatment caused estrogen levels became low so that the proliferation and cornification of vaginal epithelial cells was disrupted and omitted the estrus phase in rats [40].

The IPTS had a significantly higher serum estradiol content than the other groups (Table 2). It may be caused by the addition of tempeh in an instant cream soup. Bedell et al. [41] stated that tempeh was rich in genistein and daidzein, which were part of phytoestrogens. Phytoestrogens are polyphenolic molecules that share structural similarities and estrogenic activity with endogenous human hormones. Phytoestrogen activity was also related to the structural similarity of *β*-estradiol [41]. The similarity in the structure of genistein with endogenous estrogen indicated their ability to bind to the estrogen receptor [42]. The study of Kridawati et al. [40] showed that tempeh flour intervention resulted in a twofold increase in serum estrogen, which was the highest value compared to other interventions (tofu flour) in OV_X_ rats.

### 3.3. Malondialdehyde and Superoxide Dismutase in Experimental Animals

Menopause is the permanent cessation of menstrual periods [43]. In this phase, there was a decrease in estrogen antioxidant activity, which caused an increase in the formation of free radicals or reactive oxygen species (ROS) [44]. The process of ROS formation caused oxidative stress, which was characterized by an increase in malondialdehyde (MDA) [45]. Therefore, enzymes such as superoxide dismutase (SOD) are needed to protect cells from free radicals/ROS by catalyzing the conversion of superoxide anion radicals into oxygen molecules and hydrogen peroxide [46].

The analysis of MDA levels showed values ranging from 4.47 to 6.20 nmol mL^−1^, in which the group with OVx treatment (OH, IPS, and IPTS) had increased MDA levels (Table 3). The increase is thought to be due to the surgical procedure. Serin et al. [47] stated that surgical procedures could cause oxidative stress in the form of MDA formation. This result was similar to Sakundech et al. [48], which stated that OVx treatment caused pain stress and oxidative stress, which resulted in an increase in MDA levels in female dogs. Kridawati et al. [40] also showed that MDA levels in the serum and brain of 15-month-old rats treated with OVx were higher than those of non-OVx rat. These results were also found in blood, bone tissue, and plasma [49].

Antioxidants can reduce MDA levels in the body [50]. The intervention treatments (IPS and IPTS) showed differences in MDA levels in rat liver, in which IPTS had a lower value. The content of isoflavones in IPTS is thought to act as an antioxidant capable of neutralizing free radicals and preventing the formation of MDA due to lipid peroxidation [51]. The study of Haryati et al. [52] and Setyarini et al. [53] showed that the antioxidant in *Vigna unguiculata* was able to reduce brain and serum MDA levels after OVx treatment significantly.

SOD is a group of metalloenzymes that are the front line of defense against reactive oxygen species [54]. SOD analysis showed significant differences between groups. The OH group had the lowest SOD activity (419.35 U mL^−1^), while the IPS and IPTS groups had the highest SOD activity (497.49 U mL^−1^ and 558.88 U mL^−1^) (Table 3). Ovariohysterectomy has been shown to alter the antioxidant defense system of cells, resulting in oxidative stress caused by the accumulation of reactive oxygen species [55]. Studies have shown that SOD in liver and erythrocytes (hepatic and erythrocyte) in OVx rats decreased compared to control and OVx given rice bran containing tocopherols and anthocyanins [56]. Another study reported that SOD in the abdominal aorta in OVx rats was lower than in control rats [57].

The high SOD activity in IPS and IPTS was due to the high content of antioxidant compounds. The antioxidant activity in pumpkin cream soup was obtained from *β*-carotene content, while the antioxidant activity in pumpkin cream soup enriched with tempeh was obtained from *β*-carotene and isoflavones. Instant pumpkin cream soup contained 1.20 g/kg *β*-carotene meanwhile instant pumpkin and tempeh cream soup contained 0.65 g/kg *β*-carotene [23]. These results are similar to those of Vardi et al. [58], who showed that *β*-carotene could reduce MDA and increase SOD activity in methotrexate-induced rats. Surya et al. [59] also stated that tempeh was able to increase SOD activity in in vitro studies. Bioconversion of isoflavone glycosides into isoflavone aglycones with higher antioxidant activity in the tempeh fermentation process is thought to cause higher SOD activity [60]. In vivo, Astawan et al. [61] reported that giving Grobogan tempeh rations to rats could be reduced MDA levels and increase SOD levels in their blood.

## 4. Conclusions

Instant cream soup made from pumpkin and tempeh contained isoflavones, both genistein and daidzein, and higher antioxidant levels than pumpkin instant cream soup without the addition of tempeh. The OVx process increased MDA levels and decreased serum estradiol and SOD levels in experimental rats. Intervention with IPS and IPTS in post-OVx rat significantly increased serum estradiol in experimental rats. Meanwhile, the effect of intervention in antioxidant activity level of rats still needs more evaluation.

## Figures and Tables

**Table 1:** The results of the analysis of isoflavones and antioxidant activity.

Groups	Genistein (*μ*g 100 g^−1^)	Daidzein (*μ*g 100 g^−1^)	Antioxidants (mg AEAC 100 g^−1^)
IPS	0	0	56.25 ± 47.31^a^
IPTS	370.86	185.61 *μ*g	134.25 ± 14.35^b^

IPS: instant pumpkin cream soup; IPTS: instant pumpkin cream soup enriched with tempeh. Values are the mean ± standard deviation (SD) of the three replicates. Different letters in the same column indicate significant differences (*p* > 0.05) between treatments.

**Table 2:** Estradiol serum content on the blood of experimental rats.

Groups	Estradiol serum (*μ*g)
Control	1.902 ± 0.30^a^
OH	2.207 ± 0.37^a^
IPS	2.367 ± 0.28^a^
IPTS	3.634 ± 0.80^b^

Control: control rats with standard feed, OH: OVx rats with standard feed, IPS: OVx rats with instant pumpkin cream soup feed, and IPTS: OVx rats with instant pumpkin cream soup enriched with tempeh. Values are the mean ± standard deviation (SD) of the three replicates. Different letters in the same column indicate significant differences (*p* > 0.05) between treatments.

**Table 3:** MDA levels and SOD activity in the blood of experimental rats.

Groups	MDA level (nmol mL^−1^)	SOD activity (U mL^−1^)
Control	4.47 ± 0.10^a^	432.51 ± 36.08^a^
OH	5.85 ± 1.98^a^	419.35 ± 70.27^ab^
IPS	6.20 ± 0.50^a^	497.49 ± 59.67^bc^
IPTS	6.07 ± 1.72^a^	558.88 ± 37.67^c^

Control: control rats with standard feed, OH: OVx rats with standard feed, IPS: OVx rats with instant pumpkin cream soup feed, and IPTS: OVx rats with instant pumpkin cream soup enriched with tempeh. Values are the mean ± standard deviation (SD) of the three replicates. Different letters in the same column indicate significant differences (*p* > 0.05) between treatments.

## Data Availability

All datasets generated or analyzed during this study are available upon reasonable request from the corresponding author.
